# Interplay Between Human Gut Bacteria *Escherichia coli* and *Lactobacillus mucosae* in the Occurrence of Neuropsychiatric Disorders in Mice

**DOI:** 10.3389/fimmu.2020.00273

**Published:** 2020-02-25

**Authors:** Jeon-Kyung Kim, Kyung-Eon Lee, Sang-Ah Lee, Hyo-Min Jang, Dong-Hyun Kim

**Affiliations:** ^1^Neurobiota Research Center, Department of Pharmacy, Kyung Hee University, Seoul, South Korea; ^2^Department of Life and Nanopharmaceutical Sciences, Kyung Hee University, Seoul, South Korea

**Keywords:** neuropsychiatric disorder, inflammation, gut bacteria, brain, colon

## Abstract

To understand the roles of human gut bacteria in the occurrence of neuropsychiatric disorders, we isolated inflammatory *Escherichia coli* K1 and anti-inflammatory *Lactobacillus mucosae* from healthy human feces and examined their effects on the occurrence of altered microbiota, cognitive decline, and depression in mice. Oral gavage of *Escherichia coli* K1 caused colitis, cognitive decline, and depression in mice in the elevated plus maze, tail suspension, and forced swimming tasks. However, NK41 treatment reduced K1-induced cognitive decline and anxiety/depression. Furthermore, NK41 treatment increased K1-suppressed brain-derived neurotrophic factor (BDNF) expression and BDNF^+^/NeuN^+^ cell population and suppressed K1-induced NF-κB activation and LPS^+^/Iba1^+^ and NF-κB^+^/Iba1^+^ (microglial) cell populations in the hippocampus. NK41 treatment also suppressed K1-induced TNF-α and LPS levels in the blood and TNF-α expression, myeloperoxidase activity, NF-κB^+^/CD11c^+^ and CD11b^+^/CD11c^+^ cell populations in the colon. Furthermore, NK41 treatment decreased K1-induced colonic MUC2 expression, gut Proteobacteria population, and fecal LPS levels and modified the bacterial abundance related to polysaccharide breaking and biosynthesis. In conclusion, the overgrowth of inflammatory bacteria such as *Escherichia coli* in the gastrointestinal tract can cause neuropsychiatric disorders with gut microbiota alteration and the superiority of anti-inflammatory bacteria such as *Lactobacillus mucosae* can alleviate neuropsychiatric disorders with the attenuation of altered microbiota.

## Introduction

Bidirectional networks between the brain and gut microbiota are maintained through the hypothalamus-pituitary-adrenal (HPA) axis and microbiota-gut-brain (MGB) axis (1, 2). Exposure to external stressors, such as immobilization, stimulates the brain to secrete hormones such as corticotrophin-releasing factor, via the HPA axis, which stimulate the gut immune system and modify microbiota composition and their byproduct production (3–5). The overexpression of gut microbiota byproducts such as endotoxins disturbs gastrointestinal immune responses, which can cause the secretion of neurotransmitters such as serotonin and catecholamines to fluctuate; this results in the occurrence of systemic inflammatory diseases such as ulcerative colitis, obesity, and depression (6–9). Oral administration of the commensal bacteria *Escherichia coli*, which is excessively proliferated by 2,4,6-trinitrobenzenesulfonic acid (TNBS) or immobilization stress, and peritoneal injection of its lipopolysaccharide (LPS) cause colitis, hippocampal inflammation, cognitive decline, and anxiety in mice via altered microbiota (10, 11). LPS released from *Bacteroides fragilis*, which is abundant in the gut, have been suggested to cause Alzheimer's disease (AD) (12). A single peritoneal injection of LPS activates hippocampal astrocytes through interaction between the cells of the brain-immune interface and cytokine signals and its repeated injection activates microglia (13, 14). The peritoneal injection of LPS also suppresses brain-derived neurotrophic factor (BDNF) and cAMP response element binding protein (CREB) expression by activating the NF-κB signaling pathway (13, 15). These results suggest that altered microbiota-induced endotoxemia may cause cognitive decline and anxiety by inducing neuroinflammation in the brain.

The gut microbiota of healthy humans and animals consist of >10^11^ bacteria per gram of gut contents (16, 17). They produce toxic compounds such as LPS and peptidoglycan (PG). LPS and PG are detected by macrophages, dendritic cells, and endothelial cells that are involved in the innate immune system and then activate the biosynthesis of inflammation mediators such as tumor necrosis factor (TNF)-α and interleukin (IL)-6, resulting in the inflammation (18–20). Excessive, chronic exposure to LPS in gut microbiota may cause systemic disorders via gut inflammation, such as cognitive decline and depression. However, the suppression of gut microbiota LPS production by the probiotic *Lactobacillus plantarum* C29 alleviates LPS- or TNBS-induced colitis and cognitive decline in mice (21, 22). Gut microbiota LPS production-inhibitory *Lactobacillus brevis* OW38 also increased cognitive function in aged mice (23). Oral administration of *E. coli*, which produces a large amount of LPS, significantly increases blood LPS levels in mice while treatment with *Lactobacillus johnsonii* significantly suppresses *E. coli*-induced cognitive decline and depression (10, 11). These results suggest that regulating the balance between anti-inflammatory and inflammatory gut bacteria may be essential for the treatment of neuropsychiatric disorders.

Therefore, we isolated inflammatory *E. coli* K1 and anti-inflammatory *Lactobacillus mucosae* (formerly *Lactobacillus reuteri*) NK41 from healthy human gut microbiota and examined whether K1 could cause altered microbiota, colitis, cognitive decline, and depression in mice and whether NK41 reduced K1-induced altered microbiota, cognitive decline, and depression in mice.

## Results

### Effects of *Escherichia coli* K1 and *Lactobacillus mucosae* NK41 on the NF-κB Activation in Macrophages

**Figure 1:**
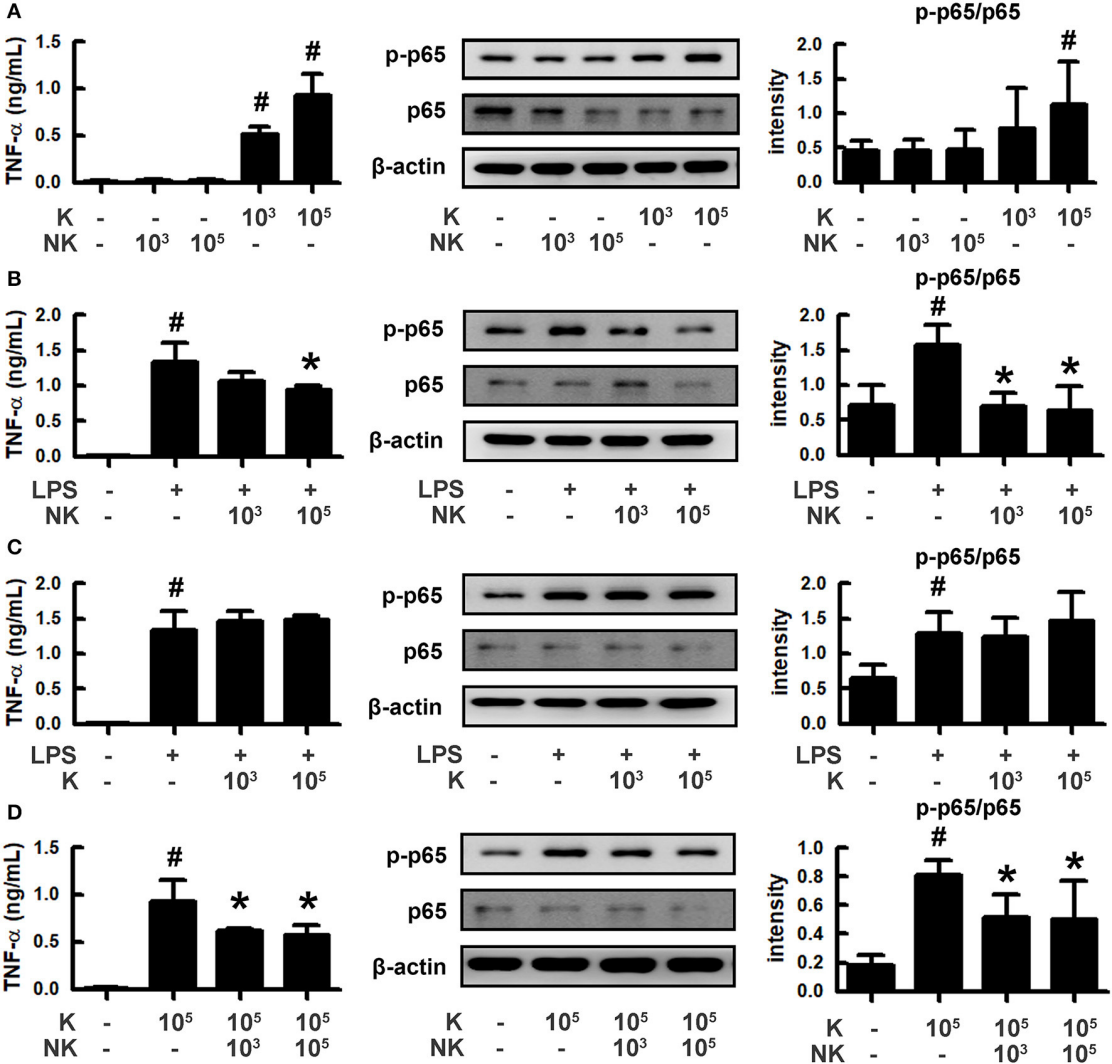
Effects of gut bacteria K1 and NK41 on the TNF-α expression and NF-κB activation in macrophages. **(A)** Effects of NK41 and K1 on TNF-α expression and NF-κB activation in macrophages. **(B)** Effects of NK41 on TNF-α expression and NF-κB activation in LPS-stimulated macrophages. **(C)** Effect of K1 on TNF-α expression and NF-κB activation in LPS-stimulated macrophages. **(D)** Effect of NK41 on NF-κB activation in K1-stimulated macrophages. Macrophage cells (1 × 10^6^/mL) were incubated with K1 or NK41 (1 × 10^3^ or 1 × 10^5^ CFU/mL) in the absence or presence of LPS for 2 h (for NF-κB) or 20 h (for TNF-α). p-p65 and p65 (NF-κB) were measured by immunoblotting. TNF-α was measured by ELISA kit. Data values are indicated as mean ± SD (*n* = 4). ^#^*p* < 0.05 vs. Con group treated with Vehicle alone. *p < 0.05 vs. group treated with K1 and LPS alone.

To understand how gut bacteria to regulate the occurrence of psychiatric disorders, we isolated gut bacteria and measured inflammatory and anti-inflammatory bacteria from human stools (Figure 1). Of these bacteria, K1 potently induced tumor necrosis factor (TNF)-α expression and NF-κB activation in macrophages, like LPS, while NK41 did not affect them. NK41 potently suppressed LPS- or K1-induced TNF-α expression and NF-κB activation in activated macrophage. Furthermore, NK41 potently hindered LPS-induced TNF-α expression in BV-2 cells (Figure S1). K1 and NK41 were identified as *E. coli* and *L. mucosae* based on the results of Gram staining, API 50 CHL Kit (bioMerieux, Seoul, South Korea), and 16S rDNA sequencing (ABI 3730XL DNA analysis), respectively.

### Effects of *Escherichia coli* K1 and *Lactobacillus mucosae* NK41 on the Occurrence of Cognitive Decline and Depression in Mice

**Figure 2:**
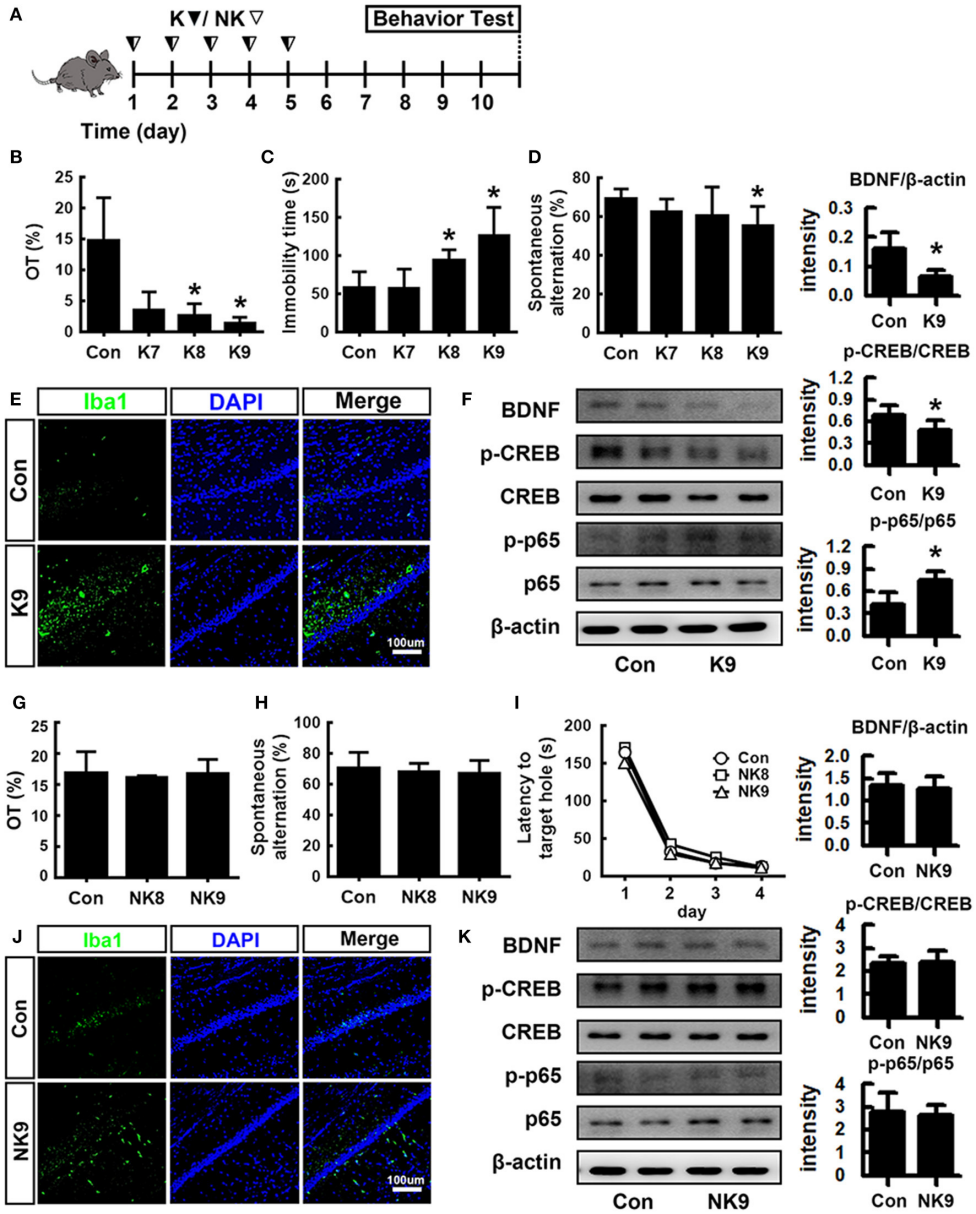
Effects of K1 and NK41 on the occurrence of neuropsychiatric disorders in mice. **(A)** Experimental protocol. **(B)** Effect of K1 on the time spent in open arms (OT) in EPM task. **(C)** Effect of K1 on the immobility in the forced swimming task. **(D)** Effect of K1 on memory impairment in Y-maze task. **(E)** Effect of K1 on the infiltration of Iba1^+^ cells into the hippocampus. **(F)** Effect of K1 on BDNF expression, CREB phosphorylation, and NF-κB activation in the hippocampus. **(G)** Effect of NK41 on the depression in EPM task. Effect of NK41 on the cogntive decline in the Y-maze **(H)** and Banes maze tasks **(I)**. **(J)** Effect of NK41 on the infiltration of Iba1^+^ cells into the hippocampus. **(K)** Effect of NK41 on BDNF expression, CREB phosphorylation, and NF-κB activation in the hippocampus. Mice were exposed to K1 or NK41 (C, vehicle [1% maltose]; K7, 1 × 10^7^ CFU/mouse/day of K1; K8, 1 × 10^8^ CFU/mouse/day of K1; K9, 1 × 10^9^ CFU/mouse/day of K1; NK8, 1 × 10^8^ CFU/mouse/day of NK41; or NK9, 1 × 10^9^ CFU/mouse/day of NK41) daily for 5 days and thereafter treated with vehicle for 5 days. Normal control group (Con), not exposed to gut bacteria, was treated with 1% maltose instead of gut bacteria. Data values were indicated as mean ± SD (*n* = 7). *p < 0.05 vs. Con group.

To understand whether inflammatory and anti-inflammatory gut bacteria were associated with the occurrence of psychiatric disorders, we examined the effects of K1 and NK41 on the occurrence of psychiatric disorders cognitive decline and depression in mice in the Y-maze, elevated plus maze (EPM), forced swimming (FS), tail suspension (TS), and Banes maze tasks (Figure 2). K1 at doses of 1 × 10^8^ and 1 × 10^9^ colony-forming unit (CFU)/mouse/day showed significant depressive behaviors in EPM and FS tasks (Figures 2B,C). Memory impairment-like behaviors were observed after treatment with K1 at a dose of 1 × 10^9^ CFU/mouse/day in the Y-maze task (Figure 2D). K1 at a dose of 1 × 10^9^ CFU/mouse/day also increased the infiltration of Iba1^+^ cells into the hippocampus. Furthermore, K1 caused NF-κB activation in the hippocampus, while the BDNF expression and CREB phosphorylation were suppressed (Figures 2E,F and Figures S2A,B, S3). However, NK41 treatment did not affect the cognitive decline in the Y-maze and Banes maze tasks and depressive behaviors in the FS task, even at a dose of 1 × 10^9^ CFU/mouse/day (Figures 2G–I). NK41 at a dose of 1 × 10^9^ CFU/mouse/day did not affect Iba1^+^ cell population, NF-κB activation, and BDNF expression in the hippocampus (Figures 2J,K and Figures S2C,D, S3).

### *Lactobacillus mucosae* NK41 Alleviated *Escherichia coli* K1-Induced Altered Microbiota in Mice

**Figure 3:**
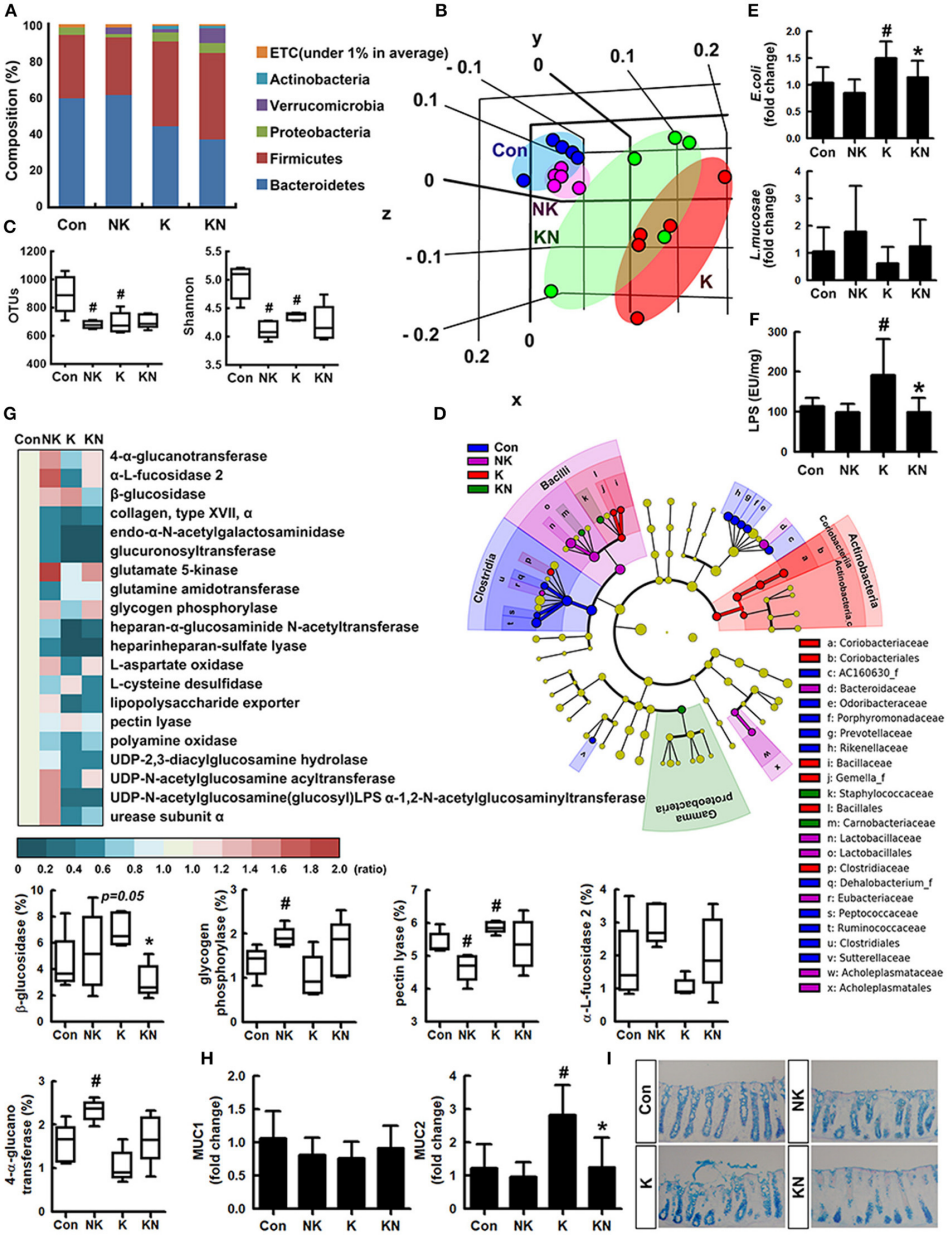
NK41 suppressed K1-induced altered microbiota in the feces mice. Effects on the composition of gut microbiota, analyzed by the pyrosequencing: phylum **(A)**, principal coordinate analysis (PCoA) plot based on weighted pairwise Fast UniFrac analysis **(B)**, and OTUs and Shannon **(C)**. **(D)** Cladogram generated by LEfSE indicating significant differences in gut microbial (family) abundances among Con (blue), NK (purple), K (red), and KN (green) groups. Yellow nodes represent species with no significant difference. The threshold logarithmic score set at 2.0 in the family level and ranked. **(E)** Effects on fecal *Escherichia coli* and *Lactobacillus mucosae*, assessed by qPCR. **(F)** Effects on the fecal LPS level. LPS levels were assayed by ELISA kits. **(G)** The abundance of bacterial genes predicted using the method of PICRUSt. The difference was analyzed using the Kruskal-Wallis H test. **(H)** Effects on the MUC1 and MUC2 expression in the colon. **(I)** Histological examination of colons, stained with alcian blue. NK and K groups were exposed to *Lactobacillus mucosae* NK41 (1 × 10^9^ CFU/mouse/day of NK41) and *Escherichia coli* K1 (1 × 10^9^ CFU/mouse/day) daily for 5 days, respectively, and thereafter treated with vehicle (1% maltose) daily for 5 days. KN group was exposed to *Escherichia coli* K1 (1 × 10^9^ CFU/mouse/day) daily for 5 days and thereafter treated with *Lactobacillus mucosae* NK41 (1 × 10^9^ CFU/mouse/day of NK41) daily for 5 days. Con group was treated with vehicle instead of gut bacteria. Data values were indicated as mean ± SD (*n* = 5). ^#^*p* < 0.05 vs. Con group. *p < 0.05 vs. K group.

To understand whether K1 and NK41 could shift gut microbiota composition, we examined their effects on the gut microbiota composition in mice (Figures 3A–C). The estimated operational taxonomic unit (OTU) richness and Shannon's diversity index were decreased in mice treated with K1 or NK41 compared to those in control mice. To match the length and position of fecal bacterial 16S rRNA gene sequences, we performed principal coordinate analysis. The bacterial community of control mouse feces was different from that of mouse ones treated with K1 or NK41. NK41 treatment similarly shifted K1-treated mouse gut microbiota to control mouse ones. At the phylum level, Proteobacteria and Actinobacteria populations showed a higher abundance in the K1-treated group compared to those in the control mouse ones, while the Bacteroidetes and Verrucomicrobioa populations showed a lower abundance. The Proteobacteria population showed a lower abundance and the Verrucomicrobia population showed a higher abundance in the NK41-treated group. Desulfovibrionaceae, Coriobacteriaceae, and Lactobacillaceae populations showed a higher abundance in the K1-treated group, while the Bacteroidaceae, AC160630_f, Helicobacteriaceae, Odoribacteriaceae, Prevotellaceae, and Rikenellaceae populations showed a lower abundance. Akkermansiaceae, Bacteroidaceae, and Lactobacillaceae populations showed a higher abundance in the NK1-treated group, while the Hellicobacteriaceae, Lachnospiraceae, Odoribacteriace, Prevotellaceae, and Rikenellaceae, Runinococcaceae populations showed a lower abundance (Table S1). At the genus level, Desulfovibrio, PAC001512_g, HM123997_g, Akkermansia, and PAC001472_g populations showed a higher abundance in the K1-treated group, while the PAC001074_g, PAC001692_g, and Oscillibacter populations showed a lower abundance. PAC001485_g, PAC000198_g, Akkermansia, and PAC001472_g populations showed a higher abundance in the NK1-treated group, while the Alistipes, PAC001692_g, Oscillibacter, Muribaculum, and PAC001112_g populations showed a lower abundance (Table S2). Furthermore, NK41 treatment shifted K1-induced gut microbiota composition to those in the control mouse one: the Proteobacteria and Bacteroidetes populations showed a lower abundance, while the Verrucomicrobia population showed a higher abundance in the K1-treated group. Linear discriminant analysis (LDA) effect size (LefSe) analysis was also performed to confirm the different effects of K1 and NK41 on gut microbiota (Figure 3D and Figures S4, S5). K1-treated mice had a higher abundance of Coridobacteriaceae, Bacillaceae, Gemella_f, and Clostridiaceae populations, while NK41 treatment resulted in a higher abundance of Bacteroidaceae, Lactobacillaceae, Eubacteriaceae, and Acholeplasmataceae populations in control mice and Staphylococcaceae, Carnobacteriaceae, and Gammaproteobacteria populations in K1-treated mice. When the fecal *E. coli* and *L. mucosae* were analyzed in the mouse feces treated with K1 and/or NK41 by using qPCR, K1 treatment significantly increased the *E. coli* population and decreased the *L. mucosae* population (Figure 3E). However, NK41 treatment significantly decreased K1-induced *E. coli* population. Furthermore, NK41 treatment suppressed K1-induced LPS production (Figure 3F).

Oral administration of NK41 and/or K1 modified the gut bacterial gene abundance related to the catabolism and anabolism of polysaccharides and fatty acids in the gut microbiota (Figure 3G). K1 treatment suppressed the abundance of gut bacterial genes related to 4-α-glucanotransferase, α-fucosidase 2, glucuronyl transferase, glycogen phosphorylase, UDP-2,3-diacylglucosamine hydrolase, and heparinheparan sulfate lyase while that related to β-glucosidase and pectin lyase was increased. However, NK41 treatment increased the K1-suppressed the abundance of gut bacterial genes related to 4-α-glucanotransferase, α-fucosidase, β-glucosidase, glycogen phosphorylase, and UDP-2,3-diacylglucosamine acyltransferase, while the β-glucosidase, pectin lyase, and urea cycle-related gut bacterial gene abundance was suppressed.

To understand whether NK41 and K1 could affect the biosynthesis of mucins such as MUC1 and MUC2 in the intestine, we examined their effects on the mucin expression in the colon (Figure 3H). K1 treatment significantly induced the expression of MUC2, not MUC1, while NK41 treatment did not affect the expression of MUC1 and MUC2. Furthermore, NK41 treatment significantly suppressed MUC1 and MUC2 expression. When the colon of mice was stained with alcian blue, the colon of K1-treated mice was strongly stained, manifested by disrupted and shortened epithelia (Figure 3I).

### *Lactobacillus mucosae* NK41 Alleviated *Escherichia coli* K1-Induced Colitis in Mice

**Figure 4:**
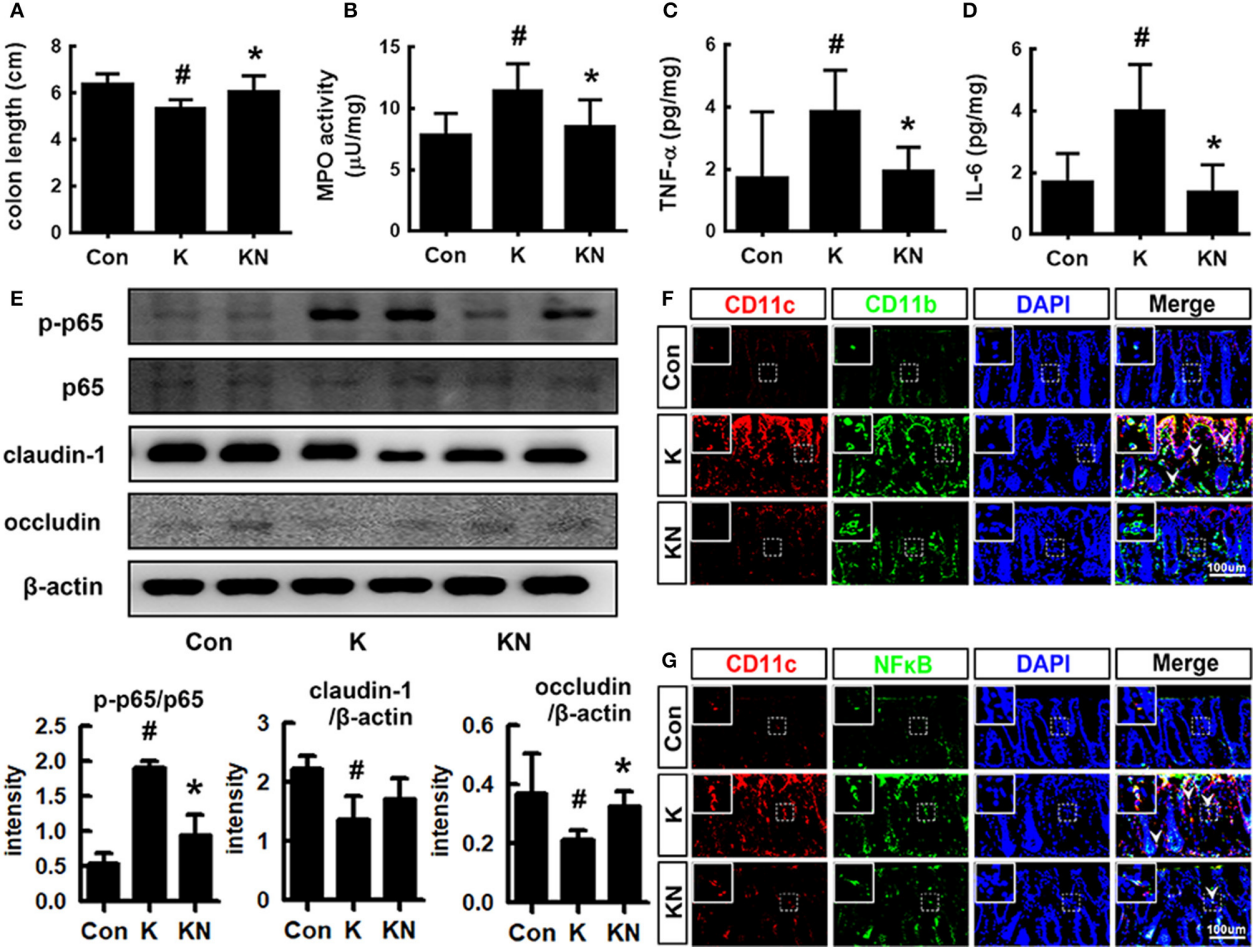
NK41 signiticantly suppressed K1-induced gut inflammation in mice. Effects on colon length **(A)**, myeloperoxidase (MPO) activity **(B)**, and TNF-α **(C)** and IL-6 **(D)** expression in the colon. **(E)** Effects on occludin and claudin-1 expression and NF-kB activation **(E)** in the colon. Effects on CD11b^+^/CD11c^+^ **(F)** and NF-κB^+^/CD11c^+^ cell populations **(G)** in the colon. NK and K groups were exposed to *Lactobacillus mucosae* NK41 (1 × 10^9^ CFU/mouse/day of NK41) and *Escherichia coli* K1 (1 × 10^9^ CFU/mouse/day) daily for 5 days, respectively, and thereafter treated with vehicle (1% maltose) daily for 5 days. KN group was exposed to *Escherichia coli* K1 (1 × 10^9^ CFU/mouse/day) daily for 5 days and thereafter treated with *Lactobacillus mucosae* NK41 (1 × 10^9^ CFU/mouse/day of NK41) daily for 5 days. Con group was treated with vehicle instead of gut bacteria. Colonic p65, p-p65, and β-actin were analyzed by immunoblotting. TNF-α and IL-6 levels were assayed by ELISA kits. NF-κB^+^, CD11b^+^, and CD11c^+^ cells were measured using a confocal microscope. Arrows indicate postive cells. Data values were indicated as mean ± SD (*n* = 7). ^#^*p* < 0.05 vs. Con group. *p < 0.05 vs. K group.

Oral gavage of K1 treatment caused colitis in mice (Figure 4). Thus, K1 treatment caused colon shortening and induced myeloperoxidase activity, IL-6 and TNF-α expression, and NF-κB activation in the colon (Figures 4A–E). Furthermore, K1 treatment increased the infiltration of NF-κB^+^/CD11b^+^ and CD11b^+^/CD11c^+^ cells (activated dendritic cells [DCs] and macrophages) into the colon (Figures 4F,G). NK41 treatment significantly reduced K1-induced colon shortening, macroscopic score, myeloperoxidase activity, IL-6, TNF-α, and MUC2 expression, NF-κB activation, and infiltration of CD11b^+^ and/or CD11c^+^ cells, while MUC1 was affected. NK41 treatment also increased the K1-suppressed claudin-1 and occludin expression (Figure 4E). Furthermore, NK41 alleviated K1-induced mucin layer damage in the colon.

### *Lactobacillus mucosae* NK41 Suppressed *Escherichia coli* K1-Induced Cognitive Decline and Depression in Mice

**Figure 5:**
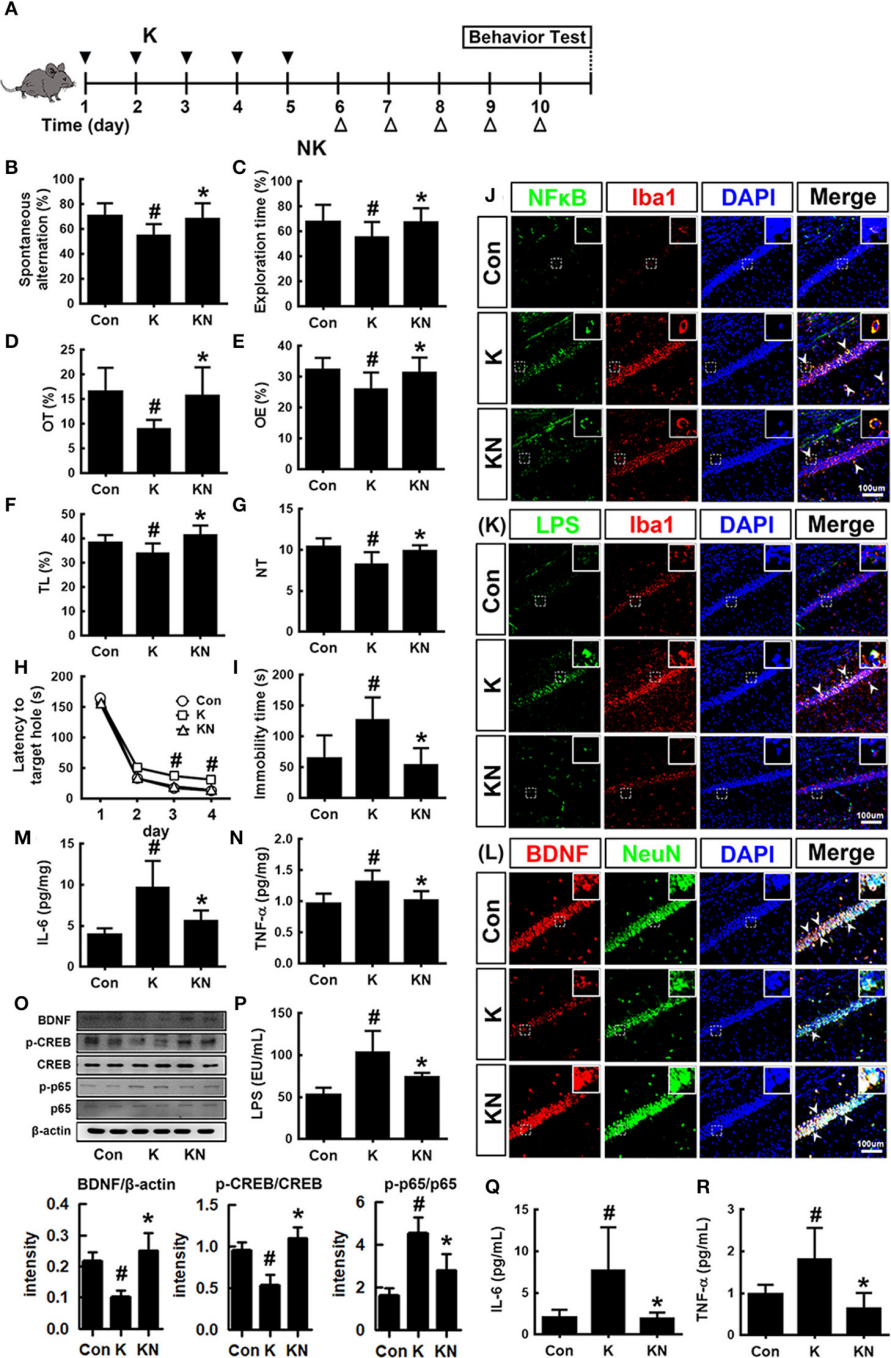
NK41 signiticantly suppressed K1-induced neuropsychiatric disorders in mice. **(A)** Experimental protocol. Effect on the cognition function in Y-maze **(B)**, NOR **(C)**, and Banes maze **(D)**. Effect on the depressive behaviors in the forced swimming **(E)**, EPM (**F**: OT, time spent in open arms; **G**: OE, open arm entries), and light-dark transition tasks (**H**: TL, time spent in the light dark compartment; **I**: NT, number of transitions into the light dark compartment). Effect on the infitration of NF-κB^+^/Iba1^+^ **(J)**, LPS^+^/Iba1^+^ **(K)**, and BDNF^+^/NeuN^+^ cells **(L)** into the hippocampus. Effects on IL-6 **(M)**, TNF-α **(N)**, and BDNF expression, CREB phosphorylation, and NF-κB activation **(O)**. Effects on the LPS **(P)**, IL-6 **(Q)**, and TNF-α levels **(R)** in the blood. NK and K groups were exposed to *Lactobacillus mucosae* NK41 (1 × 10^9^ CFU/mouse/day of NK41) and *Escherichia coli* K1 (1 × 10^9^ CFU/mouse/day) daily for 5 days, respectively, and thereafter treated with vehicle (1% maltose) daily for 5 days. KN group was exposed to *Escherichia coli* K1 (1 × 10^9^ CFU/mouse/day) daily for 5 days and thereafter treated with *Lactobacillus mucosae* NK41 (1 × 10^9^ CFU/mouse/day of NK41) daily for 5 days. Con group was treated with vehicle instead of gut bacteria. TNF-α, IL-6, and LPS were assayed by ELISA. p65, p-p65, CREB, p-CREB, BDNF, and β-actin were analyzed by immunoblotting. Iba1^+^, NF-κB^+^, LPS^+^ and NeuN^+^ cells were measured using a confocal microscope). ^#^*p* < 0.05 vs. Con group. *p < 0.05 vs. K group.

Next, we examined whether NK41 could regulate the occurrence of K1-induced psychiatric disorders in mice (Figure 5). Oral gavage of K1 caused cognitive decline in mice: its treatment significantly decreased spontaneous alteration in the Y-maze task, the interaction time in NOR task, and latency time in the Barnes maze task (Figures 5B–D). However, NK41 treatment significantly reduced K1-induced cognitive decline in Y-maze, novel object recognition (NOR) maze, and Barnes maze tasks to 98.1, 98.3, and 98.9% for the control mice, respectively. Oral gavage of K1 also caused anxiety/depression: its treatment increased immobility in the FS task to 186.1% for the control mice (Figure 5E). Furthermore, K1 treatment significantly decreased the time spent in open arms and light compartment during the EPM and light/dark transition (LDT) tasks, respectively (Figures 5F–I). However, oral administration of NK41 significantly reduced the time spent in open arms (OT) in the EPM and LDT tasks to 109.5 and 98.4% for the control mice, respectively, and immobility in the FS task to 94.6% for the control mice (Figure 5D).

K1 treatment increased the infiltration of activated/phagocytic microglial (NF-κB^+^/Iba1^+^, LPS^+^/Iba1^+^) cells into the hippocampus while the BDNF^+^/NeuN^+^ cell population was reduced (Figures 5J–L). Furthermore, K1 treatment induced NF-κB activation in the hippocampus (Figure 5O). However, treatment with NK41 suppressed K1-induced activation of NF-κB and infiltration of activated microglial cells and induced the K1-suppressed BDNF^+^/NeuN^+^ cells population in the hippocampus. NK41 treatment also suppressed K1-induced LPS, IL-6, and TNF-α levels in the blood (Figures 5P–R).

## Discussion

Excessive exposure to stressors such as immobilization, high-fat diet, and pathogen infection disrupts the gut immune system and microbiota composition through the activation of the HPA and/or MGB axis, resulting in the occurrence of altered microbiota and neuropsychiatric disorders (24–26). Long-term feeding with a high-fat diet causes obesity, colitis, and psychiatric disorders including cognitive decline and anxiety in mice by increasing the gut Proteobacteria population (27). Intrarectal injection of TNBS causes colitis and cognitive decline by increasing the gut Proteobacteria population including *E. coli* and decreasing *L. mucosae* population (10). Exposure to immobilization stress causes colitis and anxiety/depression in mice by increasing Enterobacteriaceae including *E. coli* and decreasing the populations of *L. johnsonii* and *L. plantarum* (11). Furthermore, the oral gavage of *E. coli* causes colitis, cognitive decline, and depression in mice by increasing fecal and blood LPS levels (10, 11). However, treatment with *L. mucosae*, isolated from mice, significantly alleviated *E. coli*-induced cognitive decline in mice (10). Treatment with *L. johnsonii*, isolated from mouse feces, significantly mitigated *E. coli*-induced anxiety-like behaviors in mice (11). These results suggest that gut microbiota consist of a variety of bacteria including potential causative and protective bacteria regarding neuropsychiatric disorders. Nevertheless, what kinds of gut bacteria can cause and reduce cognitive decline and anxiety/depression remain unclear.

In the present study, we isolated inflammatory *E. coli* K1, which caused NF-κB activation in macrophages, and anti-inflammatory *L. mucosae* NK41, which hindered K1 lysate- or LPS-induced NF-κB activation in macrophages. K1 significantly induced TNF-α expression in macrophages, while NK41 suppressed TNF-α expression. Furthermore, oral gavage of K1 caused colitis and hippocampal inflammation via alteration of gut microbiota in mice, resulting in cognitive decline and depression/anxiety. Exposure to K1 caused anxiety/depression as well as gut microbiota alteration that had a higher abundance of Proteobacteria and Actinobacteria populations and a lower abundance of Bacteroidetes and Verrucomicrobioa populations, while these bacterial alterations and cognitive decline and anxiety/depression were alleviated by NK41 treatment. Treatment with NK41 showed a higher abundance of Lactobacillaceae and Eubacteriaceae, and Bacteroidaceae populations. These results suggest that the overgrowth of Proteobacteria including *E. coli* K1 in the intestine by exposure to endogenous and exogenous stressors may induce cognitive decline and anxiety/depression. Oral administration of NK41 showed a lower abundance of K1-induced Proteobacteria and Enterobacteriaceae populations and LPS production in the gut microbiota of mice. These results suggest that the induction of Lactobacillaceae and Bacteroidaceae growth including *L. mucosae* NK41 can alleviate gut microbiota-mediated cognitive decline and anxiety/depression. Furthermore, NK41 treatment significantly inhibited K1-induced colon shortening, colonic myeloperoxidase activity, and infiltration of DCs and macrophages into the colon. Furthermore, NK41 suppressed the K1*-*induced expression of TNF-α and IL-6 and activation of NF-κB in the colon and increased the expression of tight junction proteins claudin-1 and occludin. NK41 treatment lowered LPS levels in the feces and blood. Jang et al. reported that fecal transplantation of IS-treated mouse feces, oral gavage of the gram-negative *E. coli* contained in it, and peritoneal injection of its LPS caused colitis: they induced myeloperoxidase activity and suppressed tight junction protein expression in the colon (11). They also found that *E. coli* treatment increased the absorption of orally administered fluorescein isothiocyanate-labeled dextran into the blood of mice. These results suggest that *E. coli* K1 can induce the excessive LPS production in gut microbiota, which leads to gut inflammation, resulting in the elevation of blood LPS by increasing gut membrane permeability. We also found that NK41 restrained K1-induced blood TNF-α and IL-6 levels in mice. NK41 also reduced K1-induced activated/phagocytic microglial (Iba1^+^) cell populations in the hippocampus. Furthermore, NK41 suppressed K1*-*induced TNF-α, IL-6, and MUC2 expression and NF-κB activation in the colon and increased K1-suppressed BDNF expression and CREB phosphorylation in the hippocampus. Moreover, NK41 treatment simultaneously improved K1-induced cognitive decline and depression in mice.

IL-6, TNF-α, and corticosterone are highly expressed in patients with anxiety and depression (28, 29). Excessive IL-6 and corticostrone expression was increased by stressors such as immobilization and pathogen infection via the activation of the HPA axis. Treatment with therapeutic drugs for psychiatric disorders reduces blood IL-6 and corticosterone levels, increases BDNF expression, and alleviates neuropsychiatric disorders (10, 11, 30). Treatment with corticosterone suppresses BDNF expression in SH-SY5Y cells *in vitro* and in mice. BDNF induces *de novo* synthesis of proteins such as synaptophysin and drebrin, which are involved in neural and synaptic plasticity (31, 32). Additionally, systemic exposure to LPS activates microglia and increased expression of pro-inflammatory cytokines in the brain of mice (10, 11). We also found that oral gavage of *E. coli* in mice caused endotoxemia and hippocampal inflammation. Therefore, *E. coli*-induced endotoxemia may cause inflammation in the brain including the hippocampus. LPS causes systemic neuroinflammation, resulting in memory impairment by the modulation of NF-κB-mediated BDNF/CREB expression (15, 33). Therefore, regulating endotoxemia-mediated corticosterone and BDNF expression can be useful for the treatment of psychiatric disorders.

We also found that *E. coli* K1 treatment significantly increased the expression of MUC2, not MUC1, and mucin layer in the colon while the colonic epithelia were disrupted. K1 treatment also increased the gut bacterial gene abundance related to polysaccharide breaking and biosynthesis. MUC1 and MUC2 are increased in patients and mice with inflammation (34, 35). LPS from gram-negative *Pseudomonas aeruginosa* upregulates MUC2 transcription through activation of NF-κB (34). *E. coli* K1 treatment caused NF-κB activation in the gut and brain and increased blood LPS levels, as previously reported in mice treated with IS or LPS (10, 11). These results suggest that, although the biosynthesis of mucins such as MUC2 is accelerated, the overgrowth of *E. coli* can increase gut bacterial LPS production and cause colitis that the speed of mucosal repair does not be overcome. Gut bacteria-induced inflammation can cause systemic inflammation including hippocampal inflammation due to the increase of LPS in the blood via increased gut membrane permeability. *L. mucosae* NK41 restrained altered microbiota-induced bacterial LPS production, blood LPS levels, and hippocampal inflammation and brought about BDNF expression and CREB expression. Neuro-inflammation decreases the expression of BDNF in the mouse hippocampus, and reduced hippocampal BDNF is associated with memory and learning deficiencies (33, 36). BDNF is influenced by the gut microbiota (33). However, oral administration of *L. plantarum* and *Bifidobacterium infantis* reduced anxiety-like behavior by restoring noradrenaline levels and protecting gut microbiota alteration, respectively (37, 38). *Bifidobacterium adolescentis* IM38, a human gut bacterium, inhibited IS-induced anxiety by regulating the GABA_A_ receptor (39). *L. johnsonii*, a commensal gut bacterium of mice, suppressed IS-induced anxiety in mice by hindering gut microbiota LPS production (11). *Bifidobacterium breve* strain A1 prevents cognitive impairment in Alzheimer's disease (40). *L. plantarum* C29 improved cognitive function in mice and patient with mild cognitive decline by regulating NF-κB activation and inducing BDNF expression (22, 41). *L. plantarum* 299v improves cognitive functions in patients with major depression by decreasing kynurenine concentration (42). *Lactobacillus rhamnosus* (JB-1) regulates emotional behavior by the induction of GABA(Aα2) expression in the hippocampus (43). Moreover, the induction of altered microbiota by stressors such as antibiotics caused cognitive function via the MGB axis (2, 11). These results suggest that NK41 can reduce cognitive decline and depression by suppressing gastrointestinal and hippocampal inflammation via the regulation of altered microbiota and bacterial LPS production. Furthermore, the regulation of K1 and NK41 on the gut inflammation and altered microbiota should be closely associated with the occurrence of neuropsychiatric disorders. Nevertheless, further studies on the detailed memory impairment-ameliorating mechanism of NK41 and pathogenic mechanism of K1 are necessary.

**Figure 6:**
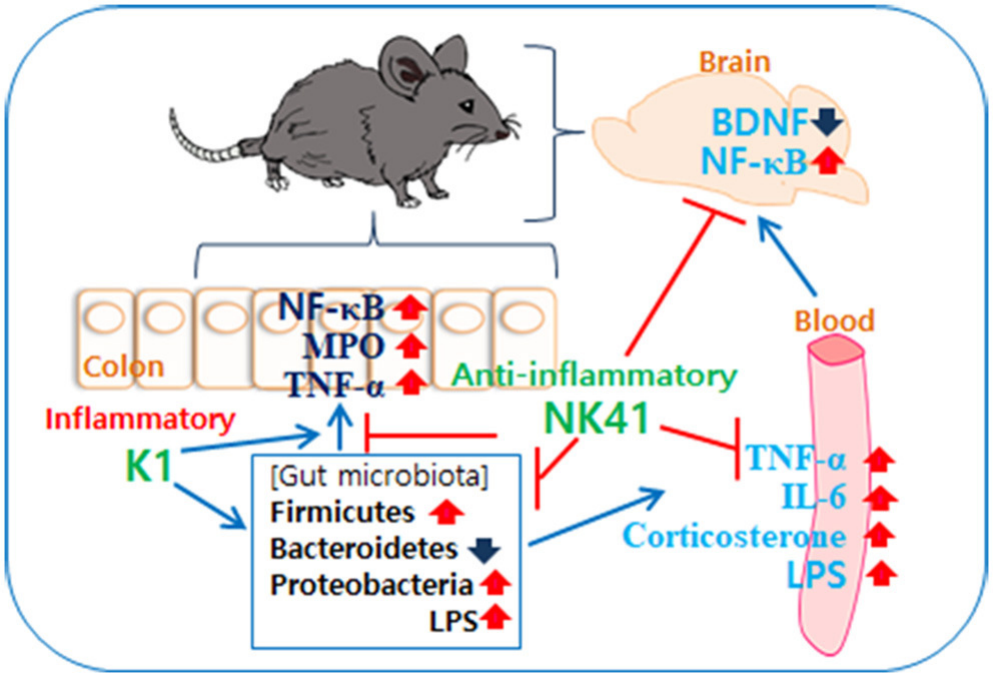
Interplay between *Escherichia coli* K1 and *Lactobacillus mucosae* NK41 on the occurrence of neuropsychotric disorders and altered microbiota.

In Conclusion, gut microbiota, which consist of inflammatory and anti-inflammatory bacteria in humans and animals, are bidirectionally connected to the brain: the overgrowth of inflammatory bacteria such as *E. coli* in the gastrointestinal tract can cause psychiatric disorders with gut inflammation and the superiority of anti-inflammatory bacteria such as *L. mucosae* can alleviate psychiatric disorders with the attenuation of altered microbiota (Figure 6).

## Methods

### Culture of *Escherichia coli* K1 and *Lactobacillus mucosae* NK41

K1 and NK41 were selected from healthy human gut microbiota according to the method of Jang et al. (10). These bacteria were cultured in general media such as general anaerobic medium (GAM) and MRS (BD, Franklin Lakes, NJ). Cultured bacteria were collected by centrifugation (5,000 g, 20 min, 4°C) and washed with saline. The collected cells were suspended in saline (for *in vitro* experiments) or 1% maltose (for *in vivo* experiments).

To decide the dosage of these bacteria in the *in vivo* experiment, K1 at doses of 1 × 10^7^, 1 × 10^8^, and 1 × 10^9^ CFU/mouse/day was orally gavaged for 5 days in mice and depression-like behaviors were measured in the EPM task, as the previously reported (44). NK41 at doses of 1 × 10^8^ and 1 × 10^9^ CFU/mouse/day was orally gavaged for 5 days in K1-treated mice and behaviors were measured in the Y-maze and elevated plus maze (EPM) tasks, as previously reported (10).

### Isolation and Culture of Peritoneal Macrophages

Macrophages were isolated from the peritoneal cavity of mice according to the method of Jang et al. (45). Collected cells were suspended in RPMI 1640 containing 10% FBS and 1% antibiotics (RFA), seeded in 6-well plate, incubated at 37°C for a day, and washed with RFA. Attached cells (1 × 10^6^ cells/well) were used as macrophages. To measure anti-inflammatory effect of gut bacteria, macrophage was treated with LPS (100 ng/mL) in the absence or presence of gut bacteria (1 × 10^3^ or 1 × 10^5^ CFU/mL) for 90 min (for p65 and p-p65) or 20 h (for TNF-α).

### Animals

Specific pathogen-free male C57BL/6J mice (19–21 g, 5 weeks-old) were purchased from Orient Inc. (Seoul, South Korea). All mice were housed in wire cages (3–4 mice per cage) at 20–22°C, 50 ± 10% humidity, and 12-h light/dark cycle (lights on from 07:30 to 19:30), fed standard laboratory chow and water *ad libitum*. Mice were used in the experiments after the acclimation for 1 week. All animal experiments were approved by The Committee for the Care and Use of Laboratory Animals in Kyung Hee University and performed in accordance with The Kyung Hee University Guidelines for Laboratory Animals Care and Usage (IACUC No. KHUASP(SE)-18089).

To examine the effects of K1 and NK41 on the occurrence and development of psychiatric disorders cognitive decline and anxiety/depression, mice were randomly divided into three groups (Control, EC, and NK groups). Each group consisted of seven mice. Mice (EC and NK groups) were orally gavaged with the K1 suspension (1 × 10^9^ CFU, suspended in 100 μL of 1% maltose) once a day for 5 days according to the method of Jang et al. (10). The control group was treated with 1% maltose instead of the K1 suspension and NK41 (for the NK group, 1 × 10^9^ CFU/mouse/day) or vehicle (for the control and EC groups) was orally administered once a day for 5 days from 24 h after treatment with K1 suspension. Behavioral tasks were performed 24 h after NK41 treatment. Mice were then anesthetized by CO_2_ asphyxiation, followed by blood draw for biochemical assays. The colon and hippocampus were removed. The specimens were stored at −80°C until use in an ELISA assay, immunoblotting, and enzyme activity assay.

### Cognitive and Depressive Behavioral Tasks

To evaluate the anti-depressive effects of gut bacteria in mice, the EPM task was performed in the plus-maze apparatus (consisting of two open [30 × 7 cm] and two enclosed arms [30 × 7 cm] with 20-cm-high walls extending from a central platform [7 × 7 cm] on a single central support to a height of 60 cm above the floor) for 5 min according to the method of Jang et al. (44). The tail suspension (TS) task was measured according to the method of Dunn and Swiergiel (46). Mice were suspended on the edge of a table 30 cm above the floor by taping 1 cm from the tail tip. Immobility time was measured for 5 min. Mice were judged to be immobile, when they did not move and hanged passively. The forced swimming (FS) task was performed in a round transparent plastic jar (20 × 40 cm^3^) containing fresh water (25°C) to a height of 25 cm. Immobility time was measured during 5 min. Mice were judged to be immobile, when they remained floating in the water without struggling.

To evaluate the cognitive effects of gut bacteria in mice, first Y-maze was performed in a three-arm horizontal maze (40 cm long and 3 cm wide with 12-cm-high walls) (10). A mouse was initially placed within one arm and the sequence and number of arm entries were manually recorded for 8 min. A spontaneous (actual) alternation was defined as entries into all three arms on consecutive choices and was calculated as the ratio (%) of actual to possible alternations. A novel object recognition (NOR) task was performed in the apparatus consisting of a dark-open field box (45 × 45 × 50 cm) according to the method of Lee et al. (47). For the first trial, a mouse was placed in the box containing two identical objects and the frequency of touching each object was recorded for 10 min. In the second trial conducted 24 h after the first trial, a mouse was placed in the box containing one of the old objects used in the first trial and a new object. Novel object recognition was calculated as the ratio of the number of times touching the new object to the sum of the touching frequencies. The Barnes maze was performed in the apparatus consisting of a circular platform (89 cm in diameter) with 20 holes (5 cm in diameter) situated evenly around the perimeter and an escape box located under only one of the holes below the platform according to the method of Patil et al. (48). The training/acquisition phase finished after mouse entered the escape box or after a maximum test duration (5 min), following which mouse was allowed to stay in the box for 30 s and then moved to the cage. If mouse failed to find the escape box within 5 min, it was led to the escape box. Mice were given two trials per day for 5 consecutive days.

### Assay of Myeloperoxidase Activity

Myeloperoxidase activity in colon was assayed, as previously described (10). Colon tissues were homogenized with cold RIPA lysis buffer and centrifuged at 10,000 *g* for 10 min (11). The supernatant was used as a crude enzyme solution. An aliquot of the supernatant was added in the reaction mixture containing 0.03% hydrogen peroxide and 1.6 mM tetramethylbenzidine and measured the absorbance at 650 nm time over 5 min. Activity was defined as the quantity degrading 1 μmol/mL of peroxide.

### Enzyme-Linked Immunosorbent Assay (ELISA) and Immunoblotting

For the cytokine assay, the supernatant of the hippocampus and colon homogenate and plasma was transferred to 96-well ELISA plates according to the method of Jang et al. (11). TNF-α concentrations were determined using commercial ELISA kits (Ebioscience, Atlanta, GA). For the immunoblotting analysis, the supernatants of tissue homogenates were resolved by sodium dodecyl sulfate polyacrylamide gel electrophoresis, transferred to nitrocellulose, and immunoblotted using various primary antibodies (Cell Signaling Technology, Beverly, MA) (11). Band densities were analyzed using the automatic imaging analysis system, Quantity One (Bio-Rad, Hercules, CA).

### Immunohistochemistry

Mice were trans-cardiacally perfused with 4% paraformaldehyde. Their brains and colons were post-fixed with 4% paraformaldehyde for 4 h, cytoprotected in 30% sucrose solution, freezed, cut using a cryostat (Leica, Nussloch, Germany), and immunostained according to the method of Jang et al. (11). Briefly, the sections were washed with phosphate buffered saline, blocked with normal serum, incubated with antibodies for Iba1 (1:200, Abcam), LPS (1:200, Millipore), NF-κB (1:100, Cell Signaling), CD11b (1:200, Abcam), CD11c (1:200, Abcam), and/or NeuN (1:200, Millipore) overnight, and treated with the secondary antibodies for 2 h. Secondary antibodies conjugated with with Alexa Fluor 488 (1:200, Invitrogen) or Alexa Fluor 594 (1:200, Invitrogen) were then treated to visualize. Nuclei were stained with 4′,6-diamidino-2-phenylindole, dilactate (DAPI, Sigma). Immunostained tissue slices were scanned with a confocal laser microscope.

### Microbiota Sequencing

Genomic DNA was extracted from the fresh stools of five mice (not trans-cardiacally perfused with 4% paraformaldehyde for brain and colon tissue sections) using a commercial DNA isolation kit (QIAamp DNA stool mini kit), as previously reported (11, 47). Briefly, genomic DNA was extracted from the fresh stools of mice using a commercial DNA isolation kit (QIAamp DNA stool mini kit). Amplification of the genomic DNA was performed using barcoded primers, which targeted the V4 region of the bacterial 16S rRNA gene, described in Supplementary Material. Sequencing for each amplicon was performed using Illumina iSeq 100 (San Diego, CA). Predictive functional genes were analyzed using the phylogenetic investigation of communities by reconstruction of unobserved states (PICRUSt) (49). Linear discriminant analysis (LDA) analysis and cladograms were developed on family level data using LDA effect size (LefSe) on Galaxy platform (https://huttenhower.sph.harvard.edu/galaxy/) (50). Pyrosequencing reads have been deposited in the NCBI's short read archive under accession number PRJNA507690.

### Quantitative Real Time–Polymerase Chain Reaction (qPCR)

Real time PCRs for MUC1, MUC2, and GAPDH were performed on the Rotor-Gene Q® using DNA polymerase and SYBR Green I (a reaction volume, 20 μL), as previously reported (11). Primers for qPCR are described in Table S3. The normalized expression of each target gene, as for GAPDH, was calculated for all samples using Microsoft Excel.

qPCRs for *E. coli, L. mucosae*, and 16S rRNA were performed on the Rotor-Gene Q® using DNA polymerase and SYBR Green I (a reaction volume, 20 μL), as previously reported (11). Thermal cycling was performed at 95°C for 30 s by 42 cycles of denaturation at 95°C for 5 s and amplification 72°C for 30 s. Expression of genes were computed relatively to 16S rDNA, using Microsoft Excel. Primers for qPCR are described in Table S4. The normalized expression of each target gene, as for GAPDH, was calculated for all samples using Microsoft Excel.

### LPS Assay

Blood and fecal endotoxin contents were determined using the diazo-coupled limulus amoebocyte lysate (LAL) assays (Cape Cod Inc., E. Falmouth, MA) according to the method of Kim et al. [(51), Supplementary Material].

### Statistical Analysis

Experimental results are indicated as mean ± standard deviation (SD), and were statistically analyzed using one-way analysis of variance followed by a Duncan multiple range test (*p* < 0.05). Student's *t*-tests were used to compare two groups. All *p*-values are indicated in Table S5.

## Data Availability Statement

The data generated for this study are available on request to the corresponding author.

## Ethics Statement

This study was performed according to the recommendation of the Kyung Hee University Animal Ethics Committee. The animal experimental protocol was reviewed and approved by the institutional animal use committee [KHU IACUC No. KHUASP(SE)-18089] and human gut bacteria collection protocol was reviewed and approved by the institutional review board of Kyung Hee University Hospital (KMC IRB No KHUH 0922-08-A1). The patients/participants provided their written informed consent to participate in this study.

## Author Contributions

J-KK and D-HK conceived and designed experiments and wrote the manuscript. J-KK, K-EL, S-AL, H-MJ, and D-HK analyzed data. J-KK, K-EL, and H-MJ contributed reagents, materials, and analysis tools.

### Conflict of Interest

The authors declare that the research was conducted in the absence of any commercial or financial relationships that could be construed as a potential conflict of interest.
