# Supplementary material for: Interplay Between Human Gut Bacteria Escherichia coli and Lactobacillus mucosae in the Occurrence of Neuropsychiatric Disorders in Mice
Source: Front Immunol. 2020 Feb 25;11:273. doi: 10.3389/fimmu.2020.00273 (PMC7051986; doi:10.3389/fimmu.2020.00273)
Supplement: Supplementary file 1 [file Data_Sheet_1.docx]

**[Supplement]**

**Interplay between human gut bacteria *Escherichia coli* and *Lactobacillus mucosae* on the occurrence of neuropsychiatric disorders in mice**

*Culture of BV-2 cells*

Murine microglial BV-2 cells (Korea Cell Line Bank, Seoul, Korea) were cultured at 37°C in a 95% air/5% CO_2_ atmosphere in DMEM containing 5% fetal bovine serum and 1% antibiotic-antimycotic (Lee et al., 2017). For the assay of TNF-α expression, BV-2 cells (1 × 10^6^ cells/mL) were incubated with LPS (100 ng/mL) or K1 (1 × 10^5^ CFU/mL) in the absence or presence of NK41 or K1 (1 × 10^3^ or 1 × 10^5^ CFU/mL) for 20 h. TNF-α expression was assayed by enzyme-linked immunosorbent assay.

*Determination of LPS in the blood and feces*

The contents of LPS in the blood and feces were assayed by using the diazo-coupled limulus amoebocyte lysate (LAL) assays according to the method of Kim et al. (2012). For the LPS assay in the blood, bloods collected by retroorbital bleeding into ethylenediaminetetraacetic acid-coated BD Microtainer^®^ tubes (Becton Dickinson, Franklin Lakes, NJ, USA) were centrifuged at 13,200 *g* for 15 min. The supernatant (5 μL) was diluted 1:10 in pyrogen-free water and inactivated for 10 min at 70°C. For the fecal LPS assay, feces were placed in 50 mL of PBS, sonicated for 1 h on ice, and centrifuged at 400 *g* for 10 min. Supernatants were filtrated through a 0.45 μm Millipore filter, re-filtrated through a 0.22 μm filter, and inactivated at 70°C for 10 min. LPS contents of filterates and supernatants (50 μl) were assayed using LAL assay kit according to the manufacturer’s protocol.

**Elevated Plus Maze Task**

EPM task was performed according to the method of Oh et al. (2015). The plus-maze apparatus consists of two open arms (30×7 cm) and two enclosed arm (30×7 cm) with 20 cm high walls, extending from a central platform (7×7 cm) (Figure 1F). The maze made of black plexiglas was raised 50 cm above the floor in a dimly lit room (20 lux) and a video camera was suspended above the maze to record the mouse’s movements. Each mouse was individually placed at the centre of EPM, its head facing the open arm and tested for 5 min. An arm entry was defined as entry of all four paws into an arm and the behaviour of the mouse was recorded as the time spent on open or closed arms and the number of entries into open or closed arms. The percentage of time spent in open arms [(time spent in open arms/time spent in open and closed arms) ×100] and of open arm entries [(the number of open arm entries/the number of entries of open and closed arms) ×100] were calculated. The spent time in open and closed arms were 300 s/mouse. The maze was cleaned with 70% ethanol to remove residues and odours before every trial.

Table S1. The gut microbiota composition ratio at the family level in the NK, K1 and the mixture treated mice.

| **Taxon Name** | **Composition (%)** | | | |
| --- | --- | --- | --- | --- |
|  | **Con** | **NK** | **K** | **KN** |
| AC160630_f | 1.39±0.16 | 0.93±0.47 | 0.18±0.16^#^ | 0.32±0.30^#^ |
| Akkermansiaceae | 0.81±0.86 | 3.28±2.21^#^ | 1.85±3.80 | 8.31±8.54 |
| Bacteroidaceae | 3.95±3.12 | 7.35±1.82 | 1.71±1.20 | 1.04±0.66 |
| Coriobacteriaceae | 0.02±0.01 | 0.66±0.11^#^ | 1.40±0.38^#^ | 1.25±0.82^#^ |
| Desulfovibrionaceae | 0.91±0.38 | 1.02±0.60 | 4.20±1.87^#^ | 3.66±3.42 |
| Helicobacteraceae | 2.52±2.40 | 0.93±0.95 | 0.89±1.01 | 0.73±0.80 |
| Lachnospiraceae | 23.88±12.59 | 7.15±4.08^#^ | 21.21±12.62 | 23.18±10.65 |
| Lactobacillaceae | 0.37±0.19 | 21.35±9.91^#^ | 17.00±6.45^#^ | 17.91±10.63 |
| Muribaculaceae | 43.50±11.29 | 51.90±6.16 | 41.03±19.11 | 35.12±27.98 |
| Odoribacteraceae | 1.03±0.52 | 0.03±0.04^#^ | 0.03±0.02^#^ | 0.01±0.01^#,*^ |
| Porphyromonadaceae | 1.19±1.49 | 0.10±0.08 | 0.04±0.03 | 0.07±0.11 |
| Prevotellaceae | 5.06±4.08 | 0.15±0.10^#^ | 0.17±0.16^#^ | 0.13±0.14^#^ |
| Rikenellaceae | 3.41±0.91 | 0.75±0.78^#^ | 1.25±0.71^#^ | 0.54±0.37^#^ |
| Ruminococcaceae | 9.50±5.20 | 2.20±0.93^#^ | 6.27±1.96 | 5.06±2.53 |

^#^*p*<0.05 vs. Con group. ^*^*p*<0.05 vs. K group.

Table S2. The gut microbiota composition ratio at the genus level in the NK, K1 and the mixture treated mice.

| **Taxon Name** | **Composition (%)** | | | |
| --- | --- | --- | --- | --- |
|  | **Con** | **NK** | **K** | **KN** |
| Alistipes | 2.65±0.94 | 0.75±0.78^#^ | 1.25±0.70^#^ | 0.53±0.38^#^ |
| PAC001485_g | 0.09±0.02 | 5.66±1.21^#^ | 1.38±1.36 | 2.13±2.00 |
| PAC001068_g | 12.85±4.37 | 11.50±1.80 | 6.59±7.54 | 7.73±8.38 |
| Desulfovibrio | 0.25±0.15 | 1.00±0.61^#^ | 4.12±1.89^#^ | 3.61±3.42 |
| PAC001512_g | 0.54±0.21 | 0.49±0.20 | 2.41±2.11 | 0.14±0.05^#,*^ |
| KE159538_g | 5.76±5.99 | 0.38±0.25 | 2.43±3.31 | 2.37±1.41 |
| PAC000186_g | 4.25±1.15 | 3.99±1.24 | 2.53±2.08 | 3.77±3.45 |
| PAC001124_g | 0.09±0.04 | 0.54±0.51 | 2.91±2.22^#^ | 1.71±2.59 |
| PAC001074_g | 4.78±2.42 | 3.96±2.41 | 0.33±0.27^#^ | 0.10±0.10^#^ |
| PAC000664_g | 5.92±3.58 | 1.95±1.69 | 5.94±6.91 | 7.36±5.22 |
| PAC001692_g | 2.67±1.18 | 0.01±0.01^#^ | 0.02±0.02^#^ | 0^#^ |
| Oscillibacter | 3.63±3.26 | 0.09±0.05^#^ | 0.48±0.23 | 0.53±0.19 |
| Muribaculum | 3.25±1.18 | 1.39±0.37^#^ | 4.11±2.58 | 3.33±2.65 |
| PAC000198_g | 2.22±0.37 | 7.95±1.89^#^ | 2.53±1.67 | 2.31±1.42 |
| PAC001112_g | 3.44±1.39 | 1.68±0.48^#^ | 3.13±3.26 | 6.06±7.40 |
| Helicobacter | 2.52±2.40 | 0.93±0.95 | 0.89±1.01 | 0.73±0.80 |
| Bacteroides | 3.94±3.12 | 7.34±1.82 | 1.71±1.20 | 1.04±0.66 |
| HM123997_g | 0.65±0.41 | 0.03±0.01^#^ | 7.64±3.38^#^ | 1.02±0.56^*^ |
| Akkermansia | 0.81±0.86 | 3.28±2.21^#^ | 1.85±3.80 | 8.29±8.53 |
| PAC001472_g | 0.41±0.14 | 6.44±2.03^#^ | 5.78±2.75^#^ | 3.65±1.71^#^ |

^#^*p*<0.05 vs. Con group. ^*^*p*<0.05 vs. K group.

Table S3. Primers for qPCR

|  | Primer (Hasnain et al., 2010; Weng et al., 2019) | |
| --- | --- | --- |
|  | Forward | Reverse |
| Muc1 | 5'-GTCTTCAGGAGCTCTGGTGG-3' | 5'-TACCACTCCAGTCCACAGCA-3' |
| Muc2 | 5'-GTCCAGGGTCTGGATCACA-3' | 5'-CAGATGGCAGTGAGCTGAGC-3' |
| GAPDH | 5'-TGCAGTGGCAAAGTGGAGAT-3' | 5'-TTTGCCGTGAGTGGAGTCATA-3' |

Table S4. Primers for qPCR (*Escherichia coli* and *Lactobacillus mucosae*)

|  | Primer | |
| --- | --- | --- |
|  | Forward | Reverse |
| *E.coli* | 5'-CAGCCACACTGGAACTGAGA-3' | 5'-GTTAGCCGGTGCTTCTTCTG-3' |
| *L. mucosae* | 5'-GAACGCAYTGGCCCAA-3' | 5'-TCCATTGTGGCCGATCAGT-3' |
| 16s rRNA | 5'-TCGTCGGCAGCGTCAGATGTGT ATAAGAGACAGGTGCCAGCMGCCGCGGTAA-3' | 5'-GTCTCGTGGGCTCGGAGATGT GTATAAGAGACAGGGACTACHV GGGTWTCTAAT-3' |

Table S5. P values of Figures

| Figure 1 | | | | |
| --- | --- | --- | --- | --- |
| TNF-α (ng/ml) | | | | |
| (A)  F(4,15)  =62.88 | Con&NK 10^3^ CFU/mL | p=0.060 | Con&K 10^3^ CFU/mL | p=0.000 |
|  | Con&NK 10^5^ CFU/mL | p=0.145 | Con&K 10^5^ CFU/mL | p=0.000 |
| (B)  F(3,12)  = 53.91 | Con&LPS | p=0.000 | LPS&NK 10^3^ CFU/mL with LPS | p=0.128 |
|  | LPS&NK 10^5^ CFU/mL with LPS | p=0.037 |  | |
| (C)  F(3,12)  =80.34 | Con&LPS | p=0.000 | LPS&K 10^3^ CFU/mL with LPS | p=0.434 |
|  | LPS&K 10^5^ CFU/mL with LPS | p=0.303 |  | |
| (D)  F(3,12)  =39.26 | Con&K 10^5^ CFU/mL | p=0.000 | K 10^5^ &NK 10^3^ with K 10^5^ CFU/mL | p=0.030 |
|  | K 10^5^ &NK 10^5^ with K 10^5^ CFU/mL | p=0.027 |  | |
| p-p65/p65 intensity | | | | |
| (A)  F(4,15)  =2.65 | Con&NK 10^3^ CFU/mL | p=0.948 | Con&K 10^3^ CFU/mL | p=0.236 |
|  | Con&NK 10^5^ CFU/mL | p=0.899 | Con&K 10^5^ CFU/mL | p=0.044 |
| (B)  F(3,12)  =12.18 | Con&LPS | p=0.002 | LPS&NK 10^3^ CFU/mL with LPS | p=0.000 |
|  | LPS&NK 10^5^ CFU/mL with LPS | p=0.002 |  | |
| (C)  F(3,12)  =7.04 | Con&LPS | p=0.004 | LPS&K 10^3^ CFU/mL with LPS | p=0.772 |
|  | LPS&K 10^5^ CFU/mL with LPS | p=0.416 |  | |
| (D)  F(3,12)  =11.90 | Con&K 10^5^ CFU/mL | p=0.000 | K 10^5^ &NK 10^3^ with K 10^5^ CFU/mL | p=0.000 |
|  | K 10^5^ &NK 10^5^ with K 10^5^ CFU/mL | p=0.000 |  | |

| Figure 2 | | | | | | |
| --- | --- | --- | --- | --- | --- | --- |
|  | | | Con&K7 | Con&K8 | | Con&K9 |
| (B) | OT(%) F(3, 24)=14.74 | | p=0.005 | p=0.003 | | p=0.002 |
| (C) | Immobility time (s) F(3,24)=10.44 | | p=0.954 | p=0.005 | | p=0.002 |
| (D) | Spontaneous alternation (%)  F(3,24)=2.60 | | p=0.091 | p=0.262 | | p=0.009 |
| (F) | BDNF/β-actin intensity | | - | - | | p=0.006 |
|  | p-CREB/CREB intensity | | - | - | | p=0.050 |
|  | p-p65/p65 intensity | | - | - | | p=0.007 |
|  | | | Con&NK8 | | Con&NK9 | |
| (G) | OT(%) F(2,18)=0.08 | | p=0.393 | | p=0.924 | |
| (H) | Spontaneous alternation (%)  F(2,18)=0.44 | | p=0.53 | | p=0.37 | |
| (I) | Latency to  target hole (s) | 1day F(2,18)=0.01 | p=0.900 | | p=1.00 | |
|  |  | 2day F(2,18)=0.54 | p=0.359 | | p=0.707 | |
|  |  | 3day F(2,18)=1.15 | p=0.124 | | p=0.988 | |
|  |  | 4day F(2,18)=0.43 | p=0.244 | | p=0.872 | |
| (K) | BDNF/β-actin intensity | | - | | p=0.687 | |
|  | p-CREB/CREB intensity | | - | | p=0.901 | |
|  | p-p65/p65 intensity | | - | | p=0.748 | |

| Figure 3 | | | | |
| --- | --- | --- | --- | --- |
|  | | Con&NK | Con&K | K&KN |
| (C) | OTU F(3,16)=8.04 | p=0.007 | p=0.017 | p=0.786 |
|  | Shannon F(3,16)=13.50 | p=0.000 | p=0.002 | p=0.398 |
| (D) | LPS (EU/mg) F(3,16)=5.26 | p=0.190 | p=0.010 | p=0.009 |
| (E) | E.coli (fold change) F(3,16)=7.19 | p=0.169 | p=0.009 | p=0.037 |
|  | L.mucosae (fold change) F(3,16)=1.52 | p=0.294 | p=0.282 | p=0.128 |
| (F) | 4-α-glucotransferase (%) F(3,16)=7.90 | p=0.010 | p=0.074 | p=0.064 |
|  | β-glucosidase (%) F(3,16)=3.29 | p=0.588 | p=0.054 | p=0.001 |
|  | glycogen phosphorylase (%) F(3,16)=3.58 | p=0.017 | p=0.245 | p=0.113 |
|  | pectin lyase (%) F(3,16)=5.94 | p=0.014 | p=0.020 | p=0.167 |
|  | α-L-fucosidase2 (%) F(3,16)=4.05 | p=0.083 | p=0.220 | p=0.076 |
| (G) | MUC1 (fold change) F(3,16)=1.55 | p=0.163 | p=0.094 | p=0.243 |
|  | MUC2 (fold change) F(3,16)=9.49 | p=0.418 | p=0.003 | p=0.007 |

| Figure 4 | | | |
| --- | --- | --- | --- |
|  | | Con&K | K&KN |
| (A) | colon length (cm) F(2,12)=7.93 | p=0.000 | p=0.039 |
| (B) | MPO activity (μU/mg) F(2,12)=6.16 | p=0.005 | p=0.034 |
| (C) | TNF- α (pg/mg) F(2,12)=4.52 | p=0.014 | p=0.046 |
| (D) | IL-6 (pg/mg) F(2,12)=11.12 | p=0.004 | p=0.002 |
| (E) | p-p65/p65 intensity F(2,12)=63.54 | p=0.000 | p=0.000 |
|  | claudin-1/β-actin intensity F(2,12)=9.07 | p=0.002 | p=1.512 |
|  | occludin/β-actin intensity F(2,12)=4.66 | p=0.035 | p=0.002 |

| Figure 5 | | | | |
| --- | --- | --- | --- | --- |
|  | | | Con&K | K&KN |
| (B) | Spontaneous alternation (%) F(2,18)=8.62 | | p=0.000 | p=0.041 |
| (C) | Exploration time (%) F(2,18)=3.81 | | p=0.018 | p=0.026 |
| (D) | OT (%) F(2,18)=5.21 | | p=0.003 | p=0.023 |
| (E) | OE (%) F(2,18)=4.20 | | p=0.028 | p=0.042 |
| (F) | TL (%) F(2,18)=7.03 | | p=0.041 | p=0.005 |
| (G) | NT F(2,18)=6.98 | | p=0.006 | p=0.033 |
| (H) | Latency to target hole (s) | 1day F(2,18)=2.02 | p=0.396 | p=0.154 |
|  |  | 2day F(2,18)=2.14 | p=0.086 | p=0.098 |
|  |  | 3day F(2,18)=5.82 | p=0.012 | p=0.076 |
|  |  | 4day F(2,18)=14.14 | p=0.031 | p=0.037 |
| (I) | Immobility time (s) F(2,18)=8.46 | | p=0.008 | p=0.003 |
| (M) | IL-6 (pg/mg) F(2,12)=8.02 | | p=0.014 | p=0.060 |
| (N) | TNF- α (pg/mg) F(2,12)=5.27 | | p=0.006 | p=0.045 |
| (O) | BDNF/β-actin intensity F(2,12)=20.25 | | p=0.000 | p=0.000 |
|  | p-CREB/CREB intensity F(2,12)=31.08 | | p=0.000 | p=0.000 |
|  | p-p65/p65 intensity F(2,12)=23.65 | | p=0.000 | p=0.001 |
| (P) | LPS (EU/mL) F(2,12)=17.02 | | p=0.001 | p=0.002 |
| (Q) | IL-6 (pg/mL) F(2,12)=8.12 | | p=0.044 | p=0.014 |
| (R) | TNF- α (pg/mL) F(2,12)=9.05 | | p=0.039 | p=0.006 |


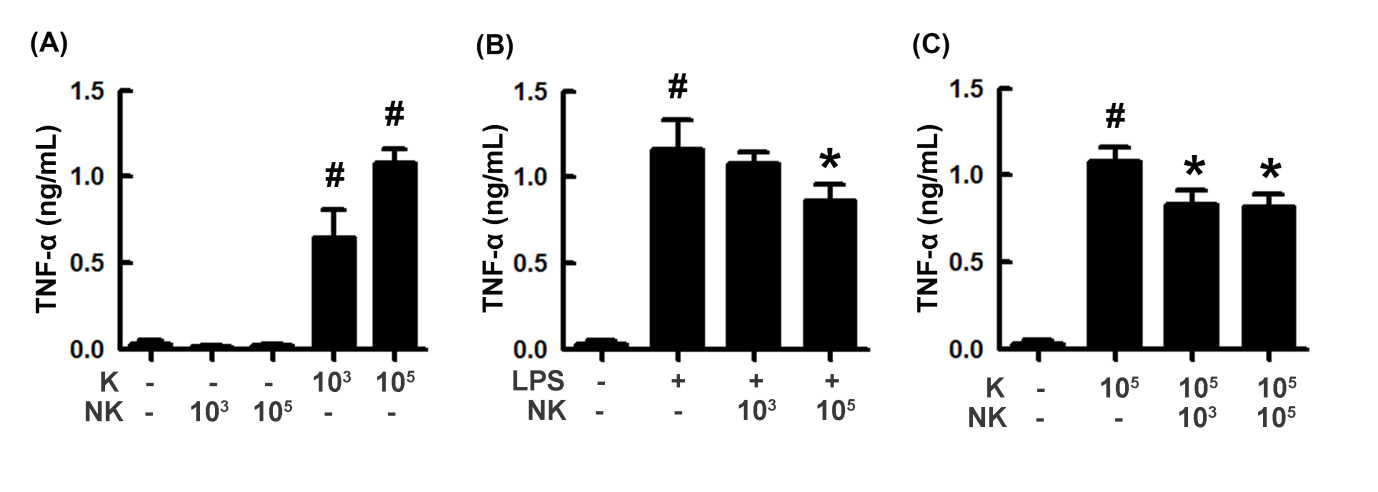


Figure S1. Effects of gut bacteria K and NK on the TNF-α expression in BV-2 cells. (A) Effects on the TNF-α expression. Cells (1 × 10^6^/mL) were incubated with K or NK (1 × 10^3^ or 1 × 10^5^ CFU/mL) for 20 h. (B) Effect of NK41 on the TNF-α expression in LPS-stimulated BV-2 cells. BV-2 cells were treated with NK (1 × 10^3^ or 1 × 10^5^ CFU/mL) in the presence of LPS (100 ng/mL) for 20 h. (C) Effect of NK41 on K1-stimuatled BV-2 cells. BV-2 cells were treated with NK (1 × 10^3^ or 1 × 10^5^ CFU/mL) in the presence of K (1 × 10^5^ CFU/mL) for 20 h. TNF-α was measured by ELISA kit. Data values are indicated as mean ± SD (n=4). ^#^p < 0.05 vs group not treated with LPS or K5; ^*^p < 0.05 vs LPS or K alone treated group.


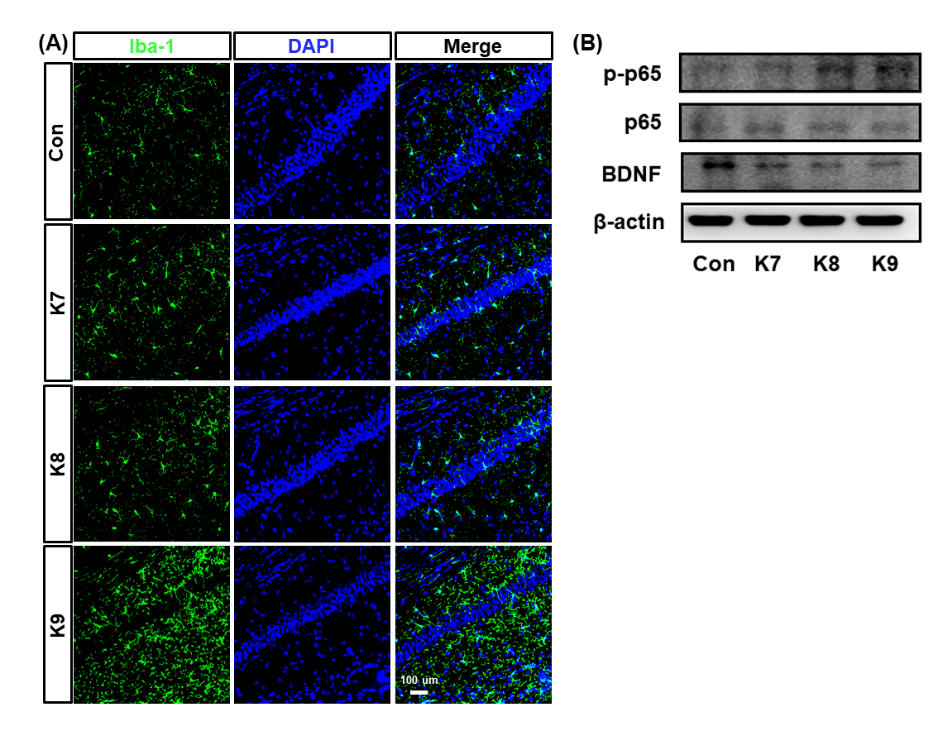


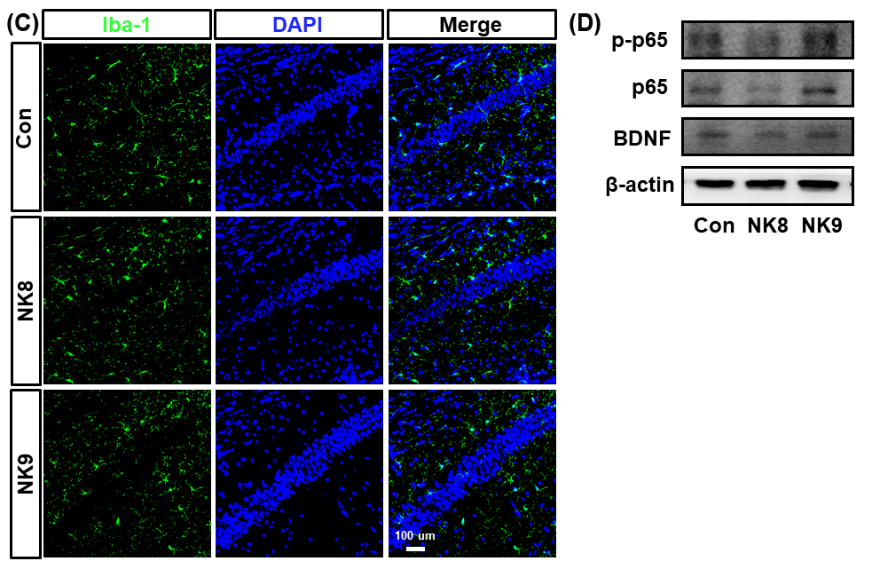


Figure S2. Effects of K1 and NK41 on the occurrence of neuropsychiatric disorders in mice. (A) Effect of K1 on the infiltration of Iba1^+^ cells into the hippocampus. (B) Effect of K1 on BDNF expression and NF-κB activation in the hippocampus. (C) Effect of NK41 on the infiltration of Iba1^+^ cells into the hippocampus. (D) Effect of NK41 on BDNF expression and NF-κB activation in the hippocampus. Mice were exposed to K1 or NK41 (C, vehicle [1% maltose]; K7, 1 × 10^7^ CFU/mouse/day of K1; K8, 1 × 10^8^ CFU/mouse/day of K1; K9, 1 × 10^9^ CFU/mouse/day of K1; NK8, 1 × 10^8^ CFU/mouse/day of NK41; or NK9, 1 × 10^9^ CFU/mouse/day of NK41) daily for 5 days and thereafter treated with vehicle for 5 days. Normal control group (Con), not exposed to gut bacteria, was treated with 1% maltose instead of gut bacteria. Data values were indicated as mean ± SD (n = 7). *p < 0.05 vs. Con group.


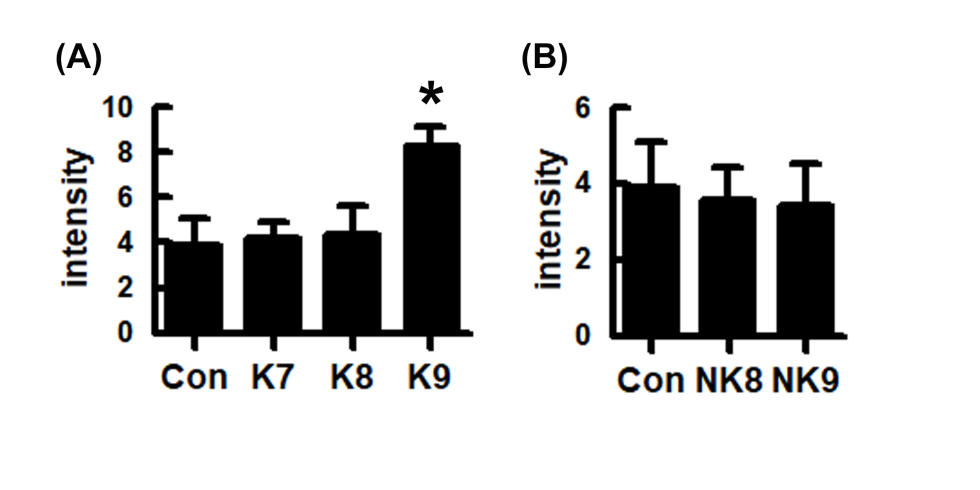


Figure S3. The intensities of Figure 2E (A) and Figure 2J (B).


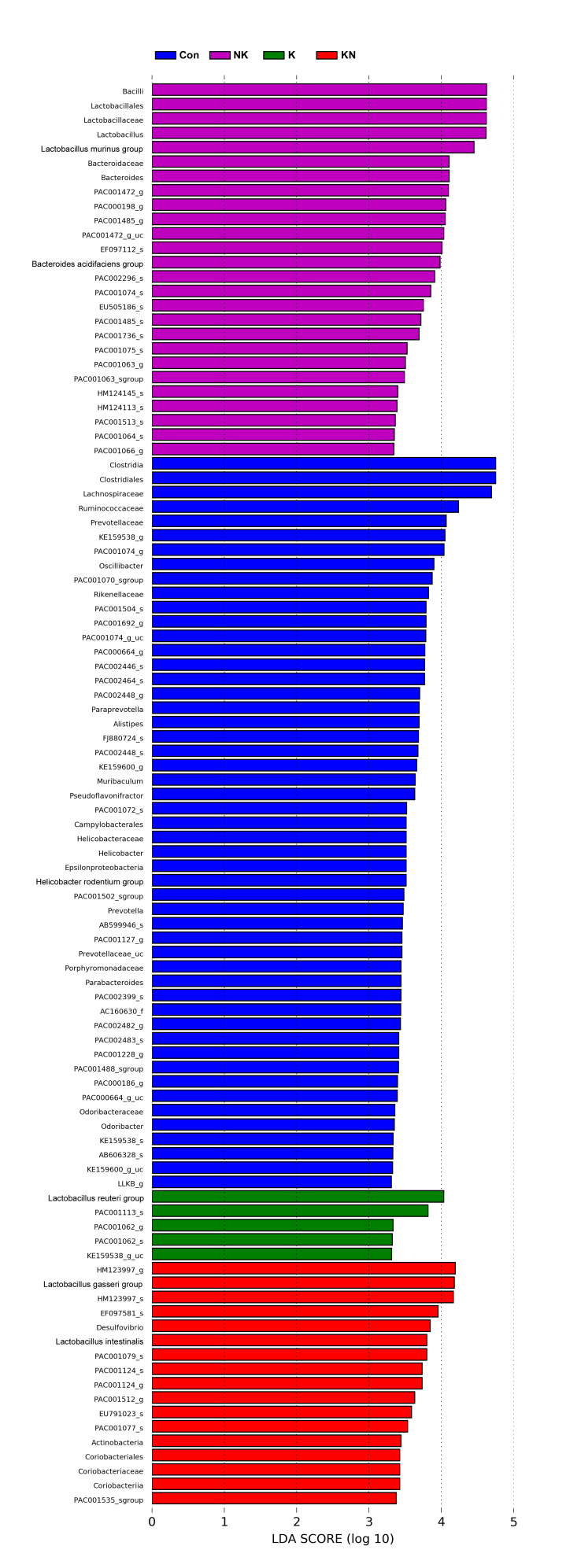


Figure S4. NK41 suppressed K1-induced gut microbiota alteration in mice. The described strains (in species) were analyzed to the Linear Discriminant Analysis (LDA) along with effect size measurement (LEfSE) in Galaxy (http://huttenhower.sph.harvard.edu/galaxy/). It was used to discriminate significant differentially strains at each taxon level. The threshold logarithmic score set at 3.3 and ranked. Bacterial strains were described based on 16SrRNA sequencing data.


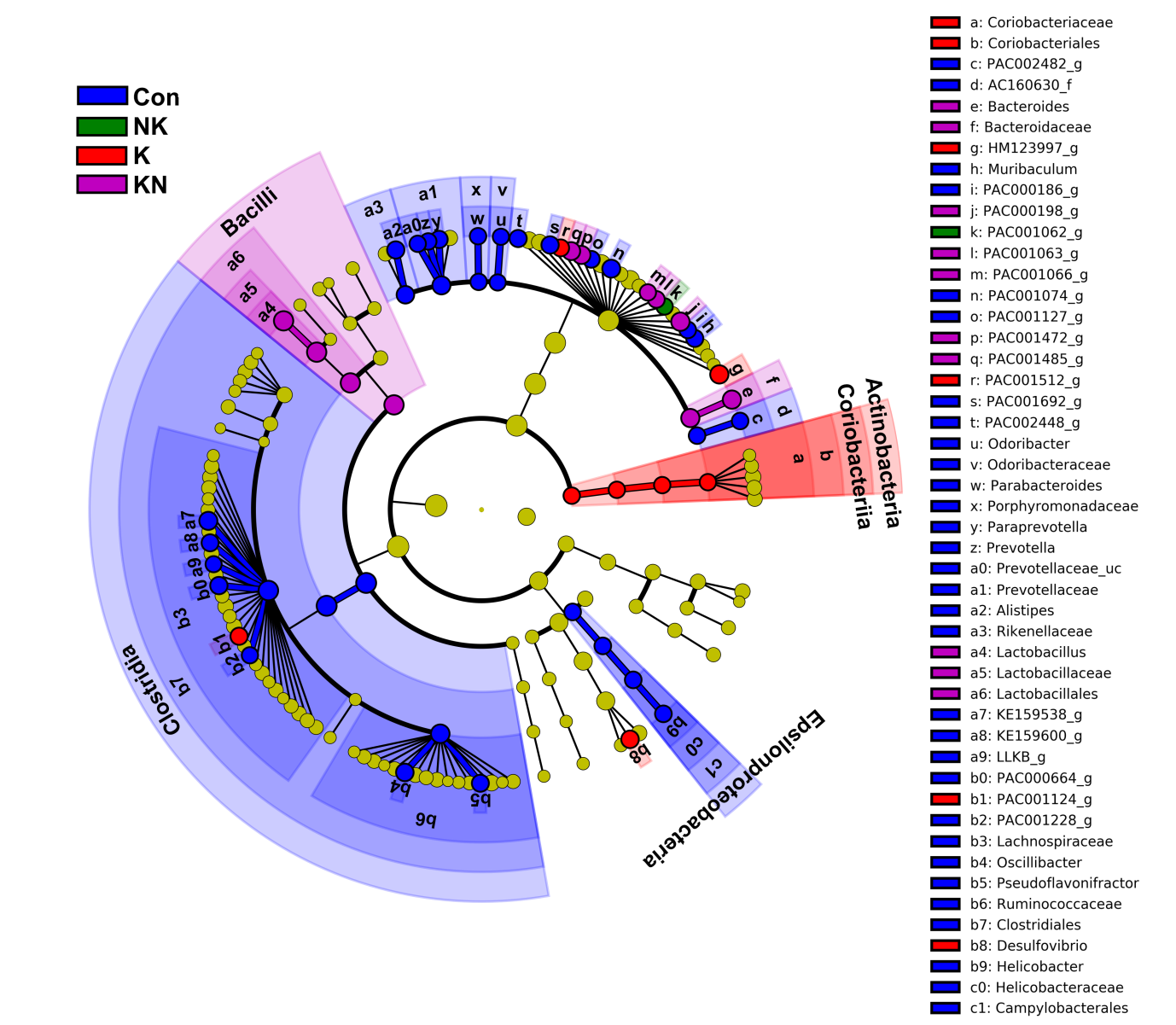


Figure S5. NK41 suppressed K1-induced gut microbiota (in genus) abundance in mice. Taxonomic cladogram was generated by performing linear discriminant analysis (LDA) of effect size (LEfSe) in Galaxy (http://huttenhower.sph.harvard.edu/galaxy/). Each circle dot represents a bacterial taxon with its diameter proportional to the taxon’s relative abundance. The threshold logarithmic score set at 3.3 and ranked.

**References**

Hasnain, S.Z., Wang, H., Ghia, J.E., Haq, N., Deng, Y., Velcich, A. et al. (2010) Mucin gene deficiency in mice impairs host resistance to an enteric parasitic infection. *Gastroenterology* 138, 1763-1771.

Kim, K.A., Gu, W., Lee, I.A., Joh, E.H. and Kim, D.H. (2012) High fat diet-induced gut microbiota exacerbates inflammation and obesity in mice via the TLR4 signaling pathway. *PLoS One* 7, e47713.

Lee, H.J., Lim, S.M., Ko, D.B., Jeong, J.J., Hwang, Y.H. and Kim, D.H. (2017) Soyasapogenol B and genistein attenuate lipopolysaccharide-induced memory impairment in mice by the modulation of NF-κB-mediated BDNF Expression. *J. Agric. Food Chem*. 65, 6877-6885.

Weng, J.S. Nakamura, T., Moriizumi,H.,Takano, H., Yao, R. and Takekawa, M. (2019) MCRIP1 promotes the expression of lung-surfactant proteins in mice by disrupting CtBP-mediated epigenetic gene silencing. *Commun. Biol*. 2, 227.
